# Does a Novel-Developed Product of Wheelchair Incorporating Pelvic Support Prevent Forward Head Posture during Prolonged Sitting?

**DOI:** 10.1371/journal.pone.0142617

**Published:** 2015-11-18

**Authors:** Hiroshi Goda, Tatsuo Hatta, Hirotoshi Kishigami, Ayaka Suzuki, Tamotsu Ikeda

**Affiliations:** 1 Graduate School of Health Sciences, Hokkaido University, Sapporo, Hokkaido, Japan; 2 Japan Health Care College, Eniwa, Japan; 3 Nihon Welfare Rehabilitation School, Eniwa, Japan; 4 Faculty of Health Sciences, Hokkaido University, Sapporo, Hokkaido, Japan; UCSD School of Medicine, UNITED STATES

## Abstract

Disabled elderly individuals with kyphosis or loss of muscle strength often display forward head posture (FHP). This study aimed to determine the utility of a wheelchair incorporating pelvic support in preventing FHP in disabled elderly individuals. In this study, 14 disabled elderly individuals (87.1 ± 8.1 years) were selected. A wheelchair incorporating pelvic support (RX_ABS Lo) and a basic wheelchair (RX-1) were used. Each individual sat on both wheelchairs for 30 minutes. RX_ABS Lo has two belts to support the pelvic and thorax. Postures were recorded in the sagittal plane using a video camera. Cervical and trunk angles from horizontal were measured every 5 minutes. Simultaneously, contact areas and total pressures applied to the wheelchair seats and back supports were measured every 5 minutes. Comparisons of area under the curve values between the wheelchairs were performed using the paired t-test. Comparisons of time-dependent parameters for each wheelchair were performed using repeated one-way ANOVA. Cervical angles were greater when using RX_ABS Lo than RX-1. Although cervical angles were unchanged during 30 minutes when using RX_ABS Lo, the angles were significantly decreased after 30 minutes of using RX-1. Back support pressures and contact areas were greater for RX_ABS Lo than for RX-1. No significant difference in back support pressure distributions was observed during 30 minutes in the wheelchairs. The RX_ABS Lo may have utility in improving FHP by increasing cervical angles and improving stability with a back support to the upper thorax, lower thorax, and pelvis during prolonged sitting.

## Introduction

Disabled elderly individuals with reduced lower limb and trunk functions often require a wheelchair for mobility and activities of daily living (ADL) [1]. The majority of wheelchair-using individuals in long-term care facilities use standard wheelchairs and are shown to have a slumped sitting posture [2, 3]. Forward head posture (FHP) is defined as a seated posture with a protracted neck and head [4, 5]. FHP is associated with fatigue and pain due to increased erector spinae load [6, 7] and vertebral disk pressure[8]. In disabled elderly individuals, FHP has been shown to increase the risk of aspiration during eating and to limit daily living[9, 10]. Exaggerated kyphosis and physical deformation resulting from decreased muscle strength are believed to be the main underlying causes of FHP in elderly wheelchair users [11, 12], along with the degree of wheelchair back support. In standard wheelchairs, a single seat sling is suspended between back support struts. This back support has been shown to increase kyphosis [13]. Elderly wheelchair users have been observed to frequently reposition themselves to ease neck pain and fatigue; however, they cannot maintain corrected postures because erect sitting increases muscle load and fatigue [14, 15].

We have developed some novel-developed product of wheelchairs incorporating a pelvic support belt for preventing FHP. RX_ABS Lo (MIKI Co., Ltd, Nagoya, Japan) is one such wheelchair. RX_ABS Lo has a pelvic support belt and is based on the “active balance seating theory” presented by Nishimura [16]. According to the theory, an ideal head and neck posture is defined as erect. RX_ABS Lo was created to achieve this sitting posture with minimal user exertion. With an erected head and neck posture, the distance between the axis of the cervical spine flexion and the center of the gravity line of the head is shorter when compared with FHP [17]. The gravitational force on the neck is counteracted by muscle activities. Thus, in an ideal posture, the gravitational force on the neck is smaller than that in FHP. Moreover, individuals can sit balanced with minimal muscle load. Before Nishimura defined the ideal posture, the criteria for the ideal posture were not clear. To clarify a comfortable ideal posture for wheelchair users, Nishimura conducted a study on healthy individuals sitting in a NA-406D wheelchair (Nissin Medical Industries Co, Ltd). This wheelchair has adjustable support belts. Using this wheelchair, Nishimura found that comfortable wheelchair sitting involves an erect head and neck posture and determined optimal support angles for the thoracic, lower thoracic, and pelvic regions to attain this posture. Support angles were reported as 22°, 35°, and approximately 19° to vertical for the thoracic, lower thoracic, and pelvic regions, respectively [16]. The setting of the support angle is considered standard in this study. RX_ABS Lo has a back support structure that supports the upper thorax, lower thorax, and pelvis to maintain the neck in an erect position.

In a previous study, we demonstrated rapid improvements of FHP in healthy elderly individuals through the use of a wheelchair incorporating a pelvic support belt [18]. However, as elderly disabled individuals have lower physical function than healthy elderly individuals, we were unable to fully evaluate the utility of wheelchairs with a pelvic support belt in preventing FHP and improving neck alignment in elderly individuals in a real-life setting. Moreover, since elderly disabled individuals use wheelchairs as chairs for daily activities, it is important to prevent FHP during prolonged sitting in wheelchairs. The present study aimed to assess the utility of a wheelchair incorporating a pelvic support belt to prevent FHP in disabled elderly individuals during prolonged sitting.

## Materials and Methods

### Individuals and ethics statement

We determined two inclusion criteria: individuals should reside at a long-term care facility in Hokkaido and understand the purpose of this research. Elderly disabled individuals residing at long-term care facilities are required to be authorized to access long-term care insurance (LTCI) [19, 20]. The criterion for applying for LTCI is that assistance is needed in daily life. The level of care severity is divided into five grades. Elderly individuals require assistance to perform daily activities in order to be authorized at a higher level.

Fourteen disabled elderly individuals (mean age, 87.1 ± 8.1 years) were selected. The mean height of all participants was 157.3 ± 12.9 cm, the mean weight was 52.1 ± 6.9 kg, and the mean daily duration of wheelchair use was 5.7 ± 3.7 hours. In terms of LTCI (Kaigo hoken [19, 20]), four individuals were at level 3 and 10 individuals were at level 4. Disabled individuals at level 3 or higher cannot walk unaided and are totally reliant on care. The estimated total duration of care required per day is 90 minutes or less for level 3 individuals and 120 minutes or less for level 4 individuals.

The individuals were selected by a collaborator of this study in their role as a care leader. The individual shown in photographs provided written informed consent (as outlined in the PLOS consent form) for these case details to be published.

Ethical approval was obtained from the Ethics Review Committee, Faculty of Health Sciences, Hokkaido University (13–85) and written informed consent was obtained prior to testing.

### RX_ABS Lo and RX-1

**Fig 1:**
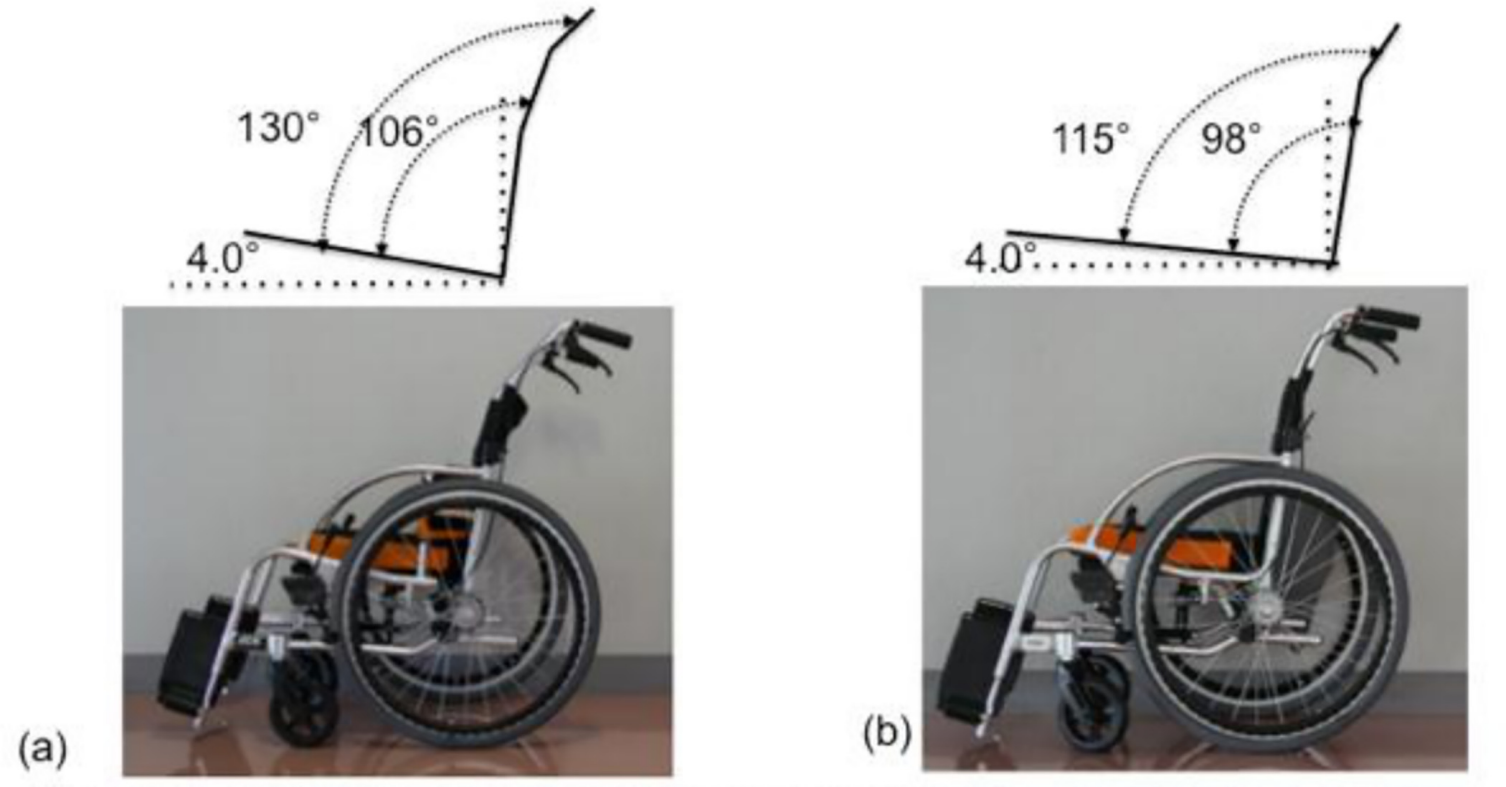
Two types of wheelchair we used in this experiment (sagittal plane). The area and height between RX_ABS Lo (a) and RX-1 (b) are same. The pipes of back support of both wheelchairs are inclined to backward in the middle of the pipe.

**Fig 2:**
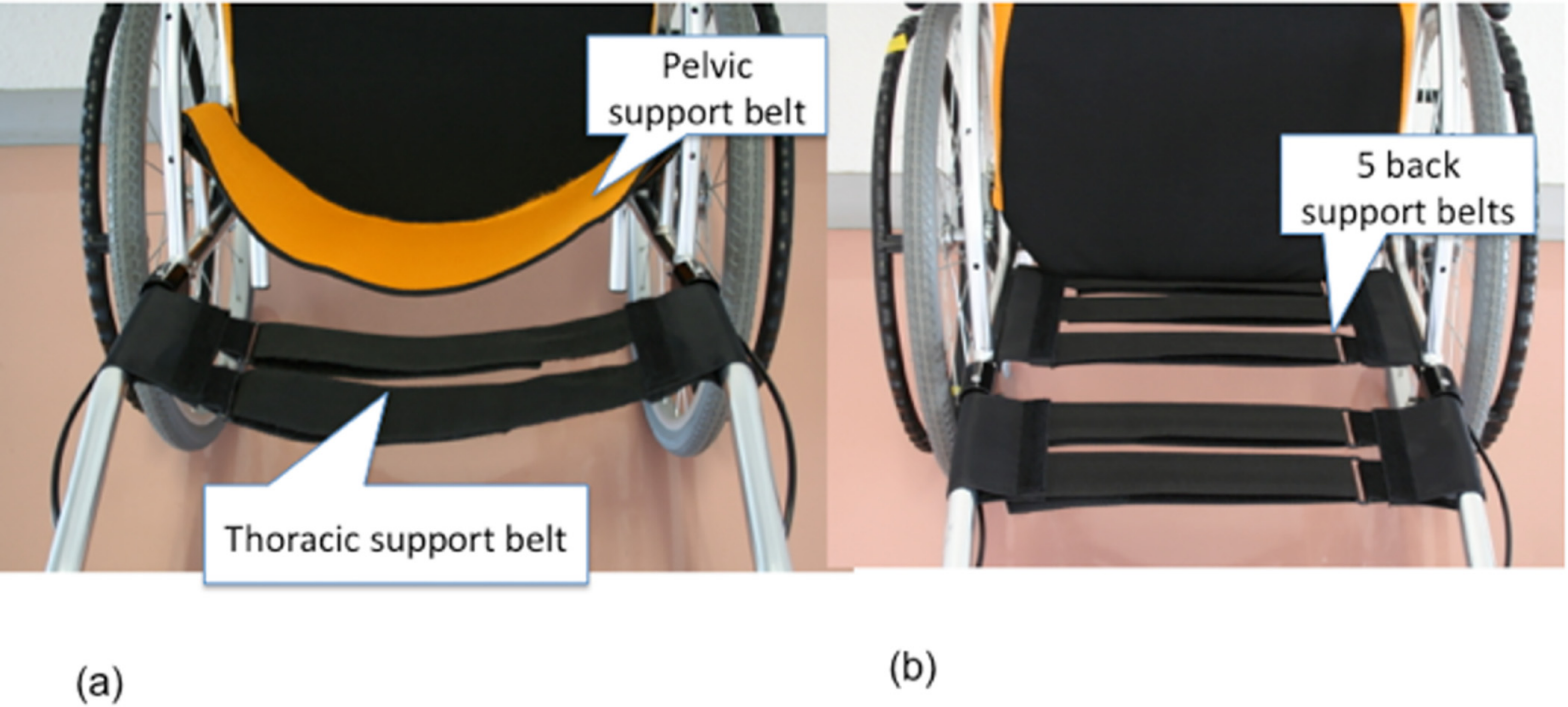
Two types of wheelchairs we used in this experiment (horizontal plane). The area and height between RX_ABS Lo (a) and RX-1 (b) are same. RX_ABS Lo has pelvic support belt and thoracic support belt (a). RX-1 has thoracic support belt. The belt was stretched tight.

Two wheelchairs were used in this study (Figs 1 and 2). RX_ABS Lo is a modification of the RX-1 (both, MIKI Co., Ltd, Nagoya, Japan). The seat surface of RX_ABS Lo is 40 cm × 40 cm. The height of the seat surface from the floor is 37.5 cm at the front and 35.0 cm at the back. The angles of the back support and armrests to the sitting surface are 106° and 130°, respectively.

ABS_RX Lo has the same common thorax support belt as RX-1. RX_ABS Lo also has a pelvic support belt. The pelvic support belt was at a height of 18 cm and took the form of an arc when viewed from above. When adjusting the belt, the posterior edge of the belt was matched to the cushion edge of the cushion when viewed from above. Velcro straps were sewn into both ends of the belt and fixed to specially designed support brackets in a twisted manner. The brackets were welded on both sides of the side guard of RX_ABS Lo. At the handles of RX_ABS Lo, two thoracic support belts were stretched across to form a ladder. The thorax support belt was stretched to 1 inch (approximately 2.5 cm) behind the pelvic support belt. As the height of the pelvic support belt was 18 cm, it could support the pelvis of the wheelchair user. The support belt is designed to accommodate pelvic rotation. The thoracic support belt provides rounded thoracic support to the wheelchair user. The setup of RX_ABS Lo was confirmed independently by three researchers.

RX-1 was used as a control because it has a structural design identical to RX_ABS Lo, except for the back support. The angles of the back support and handles to the sitting surface are 98° and 115°, respectively. Five back support belts with a width of 5 cm are arranged in the form of a ladder. Of these support belts, three are stretched between the back support struts that are vertical in relation to the sitting surface. The two remaining thoracic support belts are stretched between the wheelchair handles. The thorax support belts are identical for RX_ABS Lo and RX-1. Many wheelchairs used by disabled elderly individuals in long-term care facilities are standard wheelchairs consisting of a sling seat without an adjustable belt. Therefore, in this study, the five support belts were set at high tension to mimic standard wheelchairs. The same cushions and back covers were used for both RX_ABS Lo and RX-1.

### Beginning seating positions and individuals’ tasks

The individuals were seated with their pelvis in contact with wheelchair back supports. Positions of their pelvis were confirmed by the manual palpation of the pelvis from the front of the wheelchair. Foot support heights were set such that individuals’ thighs were parallel to the floor[15, 21] and were measured as the distance from the floor to foot supports. Foot supports were adjusted to equal heights for both wheelchairs. A historical drama that is commonly watched by elderly individuals was displayed on a computer screen placed directly in front of the individuals[22, 23]. The individuals were instructed to watch the drama in a comfortable position. Experiments were performed after confirming the stability of SR soft vision measurements. To prevent physical fatigue, RX_ABS Lo and RX-1 were experimented on a different day. Assessments were stopped when postures put the individuals at a risk of falling or when the individuals requested to end the assessments because of fatigue or pain.

### Determination of posture and number of repositioning attempts

**Fig 3:**
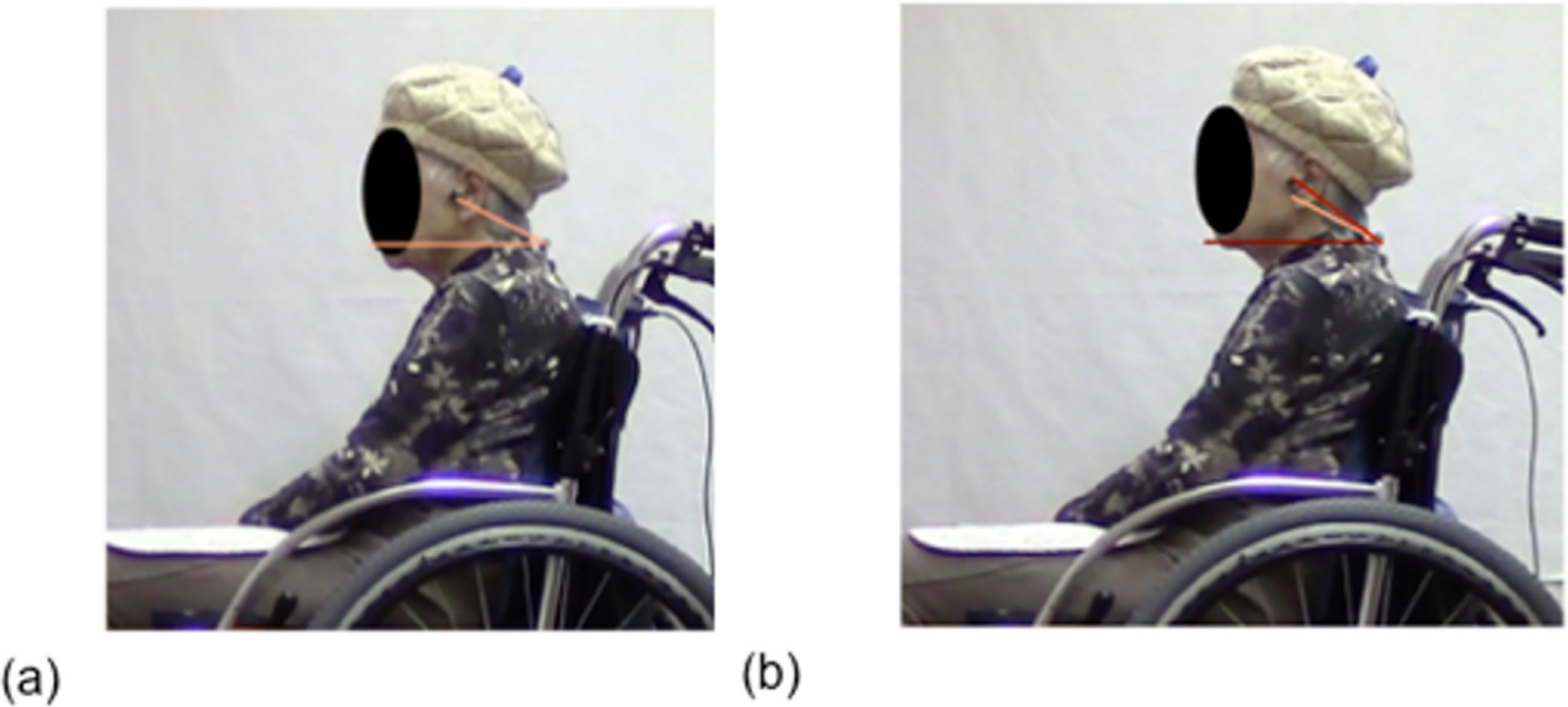
Repositioning attempts. Repositioning are defined as neck or trunk extension greater than 5°. Characteristic repositioning is shown in (a)→(b). For all individuals, there were fewer repositioning attempts with RX_RBS Lo than with RX-1 (c).

Wheelchair sitting postures were recorded using a megapixel digital camera (Canon iVIS HF M43, Japan). Surface landmarks of the neck and trunk were identified and marked by placing an adhesive reflective ball on the C7 spinous process, tragus of a ear, great trochanter, patella, and lateral malleolus of the individuals to enable posture determination. Reflective balls were adhered to the skin using a double-sided tape. Cervical and trunk angles were measured. The cervical angle was defined as the angle of the line from the tragus to the C7 spinous process and horizontal. The trunk angle was measured as the angle between the C7 spinous to great trochanter and horizontal [24–27] (Table 1). The position of each marker was recorded and automatically digitized using DARTFISH software (Dartfish Co., Ltd., Lausanne, Switzerland) [28]. For each individual, sagittal photographs of the neck and trunk were taken from the right-hand side using a megapixel digital camera fitted on a tripod located 3 m from the wheelchair [29]. The individuals affected by right-sided palsy had their postures recorded from the left-hand side [30]. To reduce expected lens errors and minimize parallax errors, the height of the camera was fixed to cover the pelvic area of the individuals at the center of the lens [29, 31, 32]. Data recorded in the sagittal plane was used to count the repositioning attempts (Fig 3). Repositioning was defined as an increase in the neck extension of more than 5° relative to the neck or trunk [33–35]. Neck extension relative to neck or trunk position was measured using DARTFISH software.

**Table 1:** Operational definitions and relevance.

	Operational definition	Relevance
**Craniocervical angle**	The angle between horizontal and the line connecting the tragus and C7 spinous process	A larger value indicates a more erect neck alignment.
**Trunk angle**	The angle between horizontal and the line connecting the C7 spinous and great trochanter	A larger value indicates an inclined trunk alignment.
**Total buttock pressure. Total Back support pressure**	The sum of measured pressures for each sensor	A larger value indicates that the mass supporting the buttocks or back is large.
**Sensor area of buttock. Sensor area of back support**	The average sensor area over 30 seconds. The area is calculated as the number of sensors activated × 4.84 cm^2^.	A larger value indicates that the buttock or back support contact area is large.
**Center of pressure in buttock pressure (right to left)**	The average length (absolute value) over 30 seconds. The length is calculated from the center of pressure to the center of the mat.	A larger value indicates a larger distance from the center of the mat.
**Center of pressure of buttock pressure (forward to backward)**	The average of the center of pressure during 30 seconds.	A larger value indicates that the center of pressure has moved backward.
**Center of pressure in back support pressure (right to left)**	The average length (absolute value) over 30 seconds. The length is calculated from the center of pressure to the center of the mat.	A larger value indicates a larger distance from the center of the mat.
**Center of pressure in back support pressure (top to bottom)**	The average center of pressure over 30 seconds.	The origin of the center of pressure is at the top of the mat. A larger value indicates that the center of pressure is at the lower portion of the mat.
**Length of left to right of the sensor area**	The length is calculated as the number of sensors × 2.2 cm.	A larger value indicates a larger transverse back support contact area.

### Measurement of back support and buttock pressures

The SR soft vision pressure mapping system (Sumitomo Riko Company Limited, Tokyo, Japan) was used to assess back support and buttock pressures. The system utilizes a 45 × 45 cm pressure mat with 256 sensors. Each sensor has an approximate width of 22 mm (area approximately 48.4 mm^2^). This system enables the measurement of pressures ranging from 20 to 200 mmHg and real-time measurement of back support and buttock pressures. The sampling frequency is 5Hz. Dedicated software was used to calculate the total pressure, area and distribution of the sensing area, location of the center of buttock and back support pressures, and length of lateral back support contact (Table 1). Two SR soft vision mats were utilized in this study. One soft vision mat was positioned to fit the posterior edge of the buttocks. Another mat was positioned to fit the lower edge of the back support.

### Data collection

Postures and pressures were measured at 0, 5, 10, 15, 20, 25, and 30 minutes. Representative values for cervical and trunk angles were measured every 5 minutes. Representative 0 minutes values for buttock and back support pressures were calculated as the average of the values from the first 30 seconds. Values at 5, 10, 15, 20, 25, and 30 minutes were calculated as the average of the values from the final 30 seconds of each period.

To estimate contact areas, still pictures were taken at 0, 5, 10, 15, 20, 25, and 30 minutes. We counted the number of contact areas in the lateral direction and multiplied it by 2.2 cm to obtain the length of the sensor. Postures were determined by viewing the recordings using QuickTime Player (Macintosh, Apple Inc.), and the timings of the individuals’ repositioning attempts with the extension of the neck or trunk were recorded. Subsequently, the number of repositioning attempts with the neck or trunk extension of more than 5° was determined using DARTFISH.

### Statistical analysis

**Fig 4:**
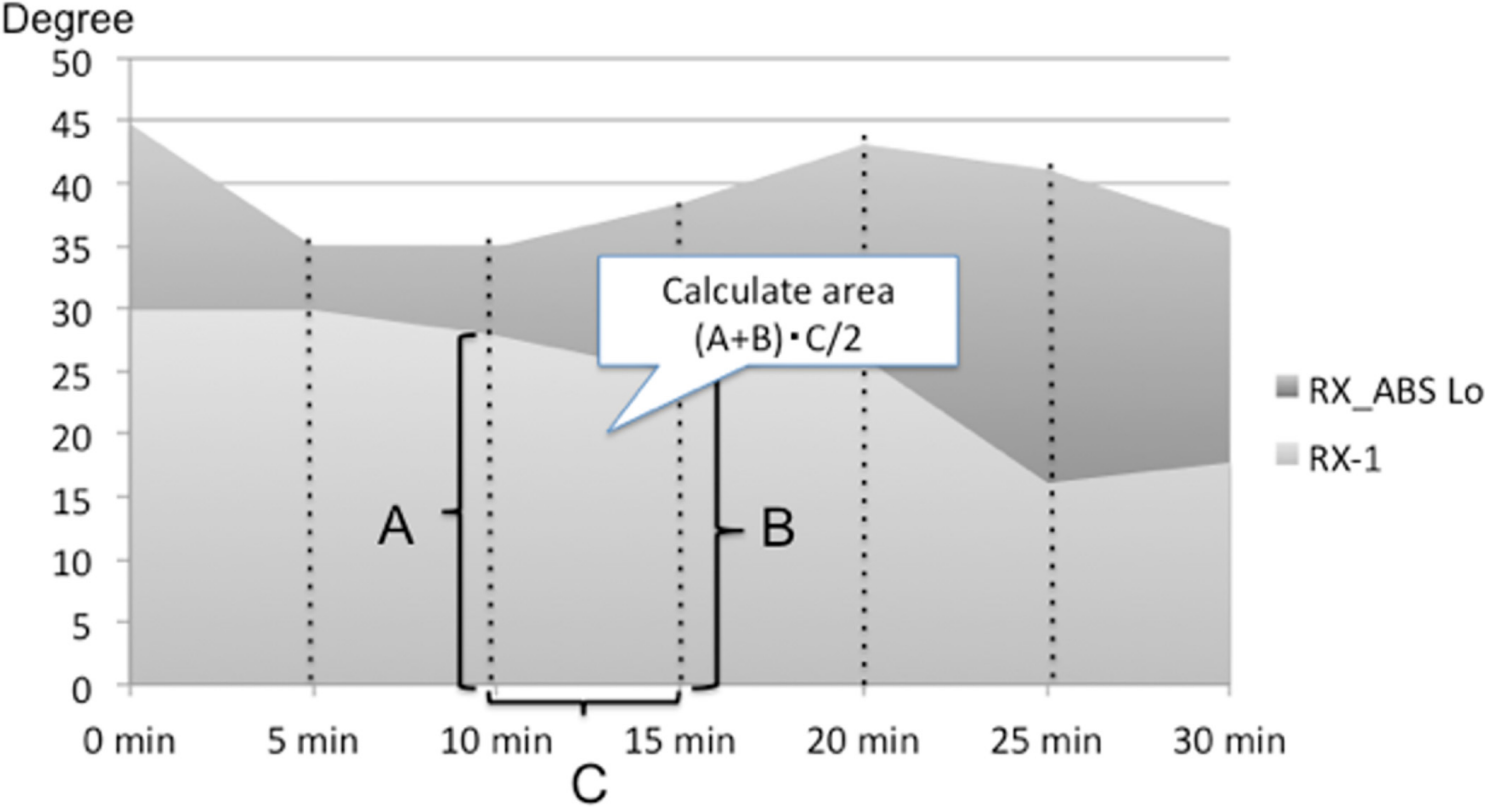
Cervical angle change in a representative subject. Time-dependent changes in cervical angle are shown. Time is represented on the horizontal axis. Six trapezia divided into 5-minute period were created to calculate the area under the curve (AUC). AUC values for RX_ABS Lo were larger than those for RX-1. These findings indicate a more erect neck alignment with RX_ABS Lo than with RX-1.

Area under the curve (AUC) values for a postural change were calculated, and a comparison of the values between RX_ABS Lo and RX-1 was performed [36]. Fig 4 shows the time dependent changes of the neck angle in RX_ABS Lo and RX-1 of an individual. AUC values were calculated from line graphs illustrating time-dependent postural changes for each wheelchair (Fig 4). Cervical angles after 10 and 15 minutes of using RX-1 are represented as “A degree” and “B degree,” respectively. In this situation, the trapezoidal area between values at 10 and 15 minutes can be calculated as (15 − 10) (A + B)/2. Representative cervical angle values for RX-1 at 30 minutes were calculated using the formula (1) as follows.

$$\frac{1}{2}\overset{6}{\underset{i=1}{\sum }}\left(x_{5i}-x_{5i-5}\right)\left(y_{5i}+y_{5i-5}\right)$$ (1)

AUC values for cervical angles, trunk angles, and pressure distributions (total buttock pressure, total back support pressure, central pressure, and lateral back support pressure length) were calculated in a similar manner for both wheelchairs. Paired t-tests were used to assess differences in posture angles and pressure distributions between RX_ABS Lo and RX-1.

Postural changes during the use of both RX_ABS Lo and RX-1 were determined using repeated one-way ANOVA. Multiple comparisons were performed using Bonferroni’s multiple comparison test with a level of significance of P < 0.05. All statistical analyses were performed using Statistical Package for the Social Sciences (version 22; IBM).

## Results

### Demographic data of individuals

Three of the initially selected 14 individuals could not complete the study. All 3 individuals requested that the assessment be stopped. One individual complained of fatigue following the first time assessment. The other two individuals also complained of fatigue during the experiment. The mean age of the 11 individuals (8 females) was 84.6 ± 7.7 years old, the mean height was 155.0 ± 11.0 cm, and the mean weight was 52.1 ± 7.6 kg. The mean duration of wheelchair use was 5.7±3.5 hours. In terms of the level of LTCI, 2 individuals were of level 3, and 9 individuals were of level 4. Disabled individuals at level 3 or higher cannot walk unaided and are totally reliant on care. The age of individuals who dropped out ranged between 87 and 98 years (the last patient to drop out was 95 years old) years old. Regarding LTCI levels, two individuals were at level 3 and one was at level 4.

### Comparison of postures and pressure distributions between RX_ABS Lo and RX-1

**Fig 5:**
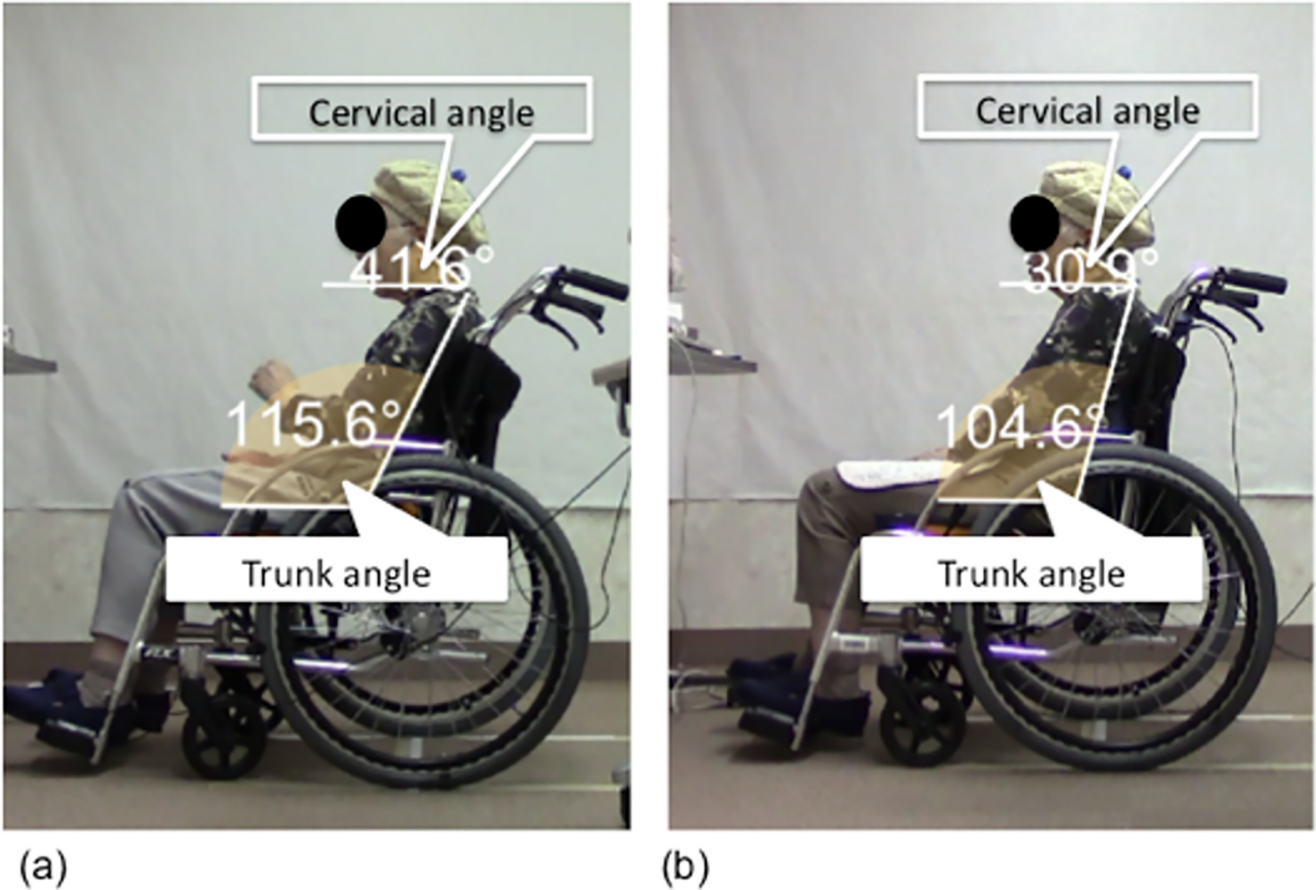
Representative sitting postures. The two wheelchair designs are identical, except for back supports. Neck alignment, measured by the cervical angle, was erect with RX_ABS Lo (a). Neck alignment was posed in a FHP with RX-1(b).

**Fig 6:**
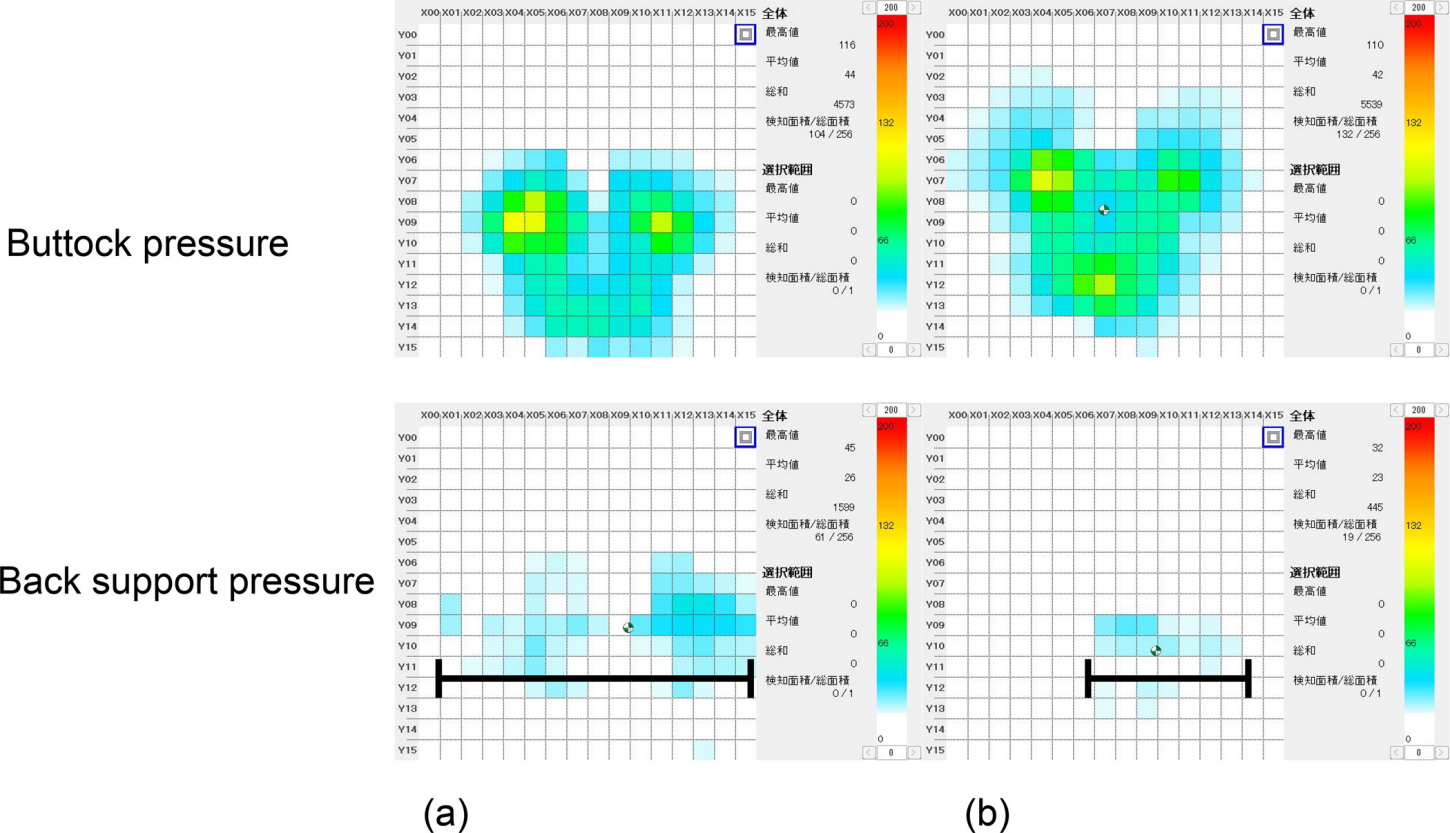
Buttock and back support pressures in a representative case. Buttock pressure with RX-ABS Lo was lower and back support pressure was higher in RX_ABS Lo than in RX-1. The back support contact area with RX_ABS Lo was greater with an increased lateral contact area (see arrow) when compared to RX-1. These findings indicate that the pelvic support belt provided adequate support for the pelvis and thorax.

A representative example of postures and back support and buttock pressure distributions with RX_ABS Lo and RX-1 is shown in Figs 5 and 6. Neck alignments with RX_ABS Lo were found to be closer to erect when compared with RX-1. Moreover, trunk angles for RX_ABS Lo had a more posterior inclination than for RX-1. Total back support pressures for RX_ABS Lo were greater than those for RX-1. Greater back support contact areas were observed with RX_ABS Lo than with RX-1.

Comparisons of AUC values for cervical and trunk angles between RX_ABS Lo and RX-1 are shown in Table 2. Cervical and trunk angles with RX_ABS Lo were greater than with RX-1 (i.e., P < 0.01 and P = 0.02, respectively). Although no significant difference in buttock pressure was observed between RX_ABS Lo and RX-1, back support pressure was greater with RX_ABS Lo than with RX-1 (P < 0.01). Greater back support contact areas were observed with RX_ABS Lo than with RX-1 (P < 0.01). The center of back support pressure was not found to differ significantly between RX_ABS Lo and RX-1.

**Table 2:** Area under the curve values for postures and pressure distributions between RX_ABS Lo and RX-1.

	RX_ABS Lo	RX-1	P-value
Craniocervical angle	987.06 ± 457.99	733.55 ± 365.05	0.00
Trunk angle	3621.27 ± 144.83	3146.31 ± 665.95	0.02
Total buttock pressure	161630.54 ± 30127.13	174422.97 ± 49278.31	0.23
Total back pressure	30274.05 ± 12521.94	18759.19 ± 10883.78	0.00
Sensor area of buttock pressure	17325.12 ± 3608.05	18300.26 ± 5605.78	0.43
Sensor area of back support pressure	5636.78 ± 2436.89	3259.79 ± 5636.78	0.00
Center of buttock pressure location (right to left)	1083.56 ± 369.50	870.09 ± 390.32	0.65
Center of buttock pressure location (forward to backward)	5732.00 ± 1038.08	6022.36 ± 1122.48	0.80
Center of back support pressure (right to left)	1032.03 ± 562.80	909.81 ± 432.94	0.82
Center of back support pressure (top to bottom)	5192.06 ± 1483.70	5596.62 ± 1572.25	0.18
Length of left to right of the sensor area	9685 ± 285.44	3044.5 ± 313.15	0.00

### Change in postures and pressure distributions in RX_ABS Lo and RX-1

Postural changes over 30 minutes use of RX_ABS Lo and RX-1 are shown in Tables 3–6. There was no significant change in cervical and trunk angles with RX_ABS Lo over time. Moreover, no significant difference in total back support pressure or center of pressure location was observed. Total buttock pressures at 10 and 15 minutes were greater than that at 5 minutes (P < 0.05). Cervical angles after 30 minutes use of RX-1 were decreased compared to 0 minutes values (P < 0.05). No significant change in trunk angle was observed with RX-1 over time. Moreover, no significant difference in buttock pressure or back support pressure was observed.

**Table 3:** Time-dependent changes in cervical and trunk angles according to the wheelchair design (RX-ABS Lo and RX-1).

	Craniocervical Angle (°)		Trunk Angle (°)	
	RX_ABS Lo	RX-1	RX_ABS Lo	RX-1
**0 min**	35.6 ± 13.3	28.1 ± 10.5	120.4±5.3	110.8±6.1
**5 min**	32.7 ± 16.8	24.4 ± 15.8	120.1±6.2	109.6±8.4
**10 min**	30.8 ± 15.8	25.3 ± 12.8	120.8±5.4	111.3±7.6
**15 min**	33.8 ± 16.2	27.1 ± 7.9	120.4±4.7	110.8±7.4
**20 min**	32.3 ± 15.0	26.7 ± 9.6	121.1±4.1	110.9±6.9
**25 min**	32.6 ± 16.7	25.0 ± 11.1	120.9±4.7	111.4±5.9
**30 min**	34.7 ± 15.1	25.0 ± 9.3	121.4± 4.7	110.4± 7.6

**Table 4:** Time-dependent changes in total buttock and back support pressures according to the wheelchair design (RX-ABS Lo and RX-1).

	Total buttock pressure (mmHg)		Total Back support pressure (mmHg)	
	RX_ABS Lo	RX-1	RX_ABS Lo	RX-1
**0 min**	5162.7 ± 877.2	5954.4 ±1143.3	1203.7± 533.6	665.3 ± 404.3
**5 min**	5224.5 ±826.3	5923.8 ±1164.7	1047.2 ±543.5	619.4 ± 411.2
**10 min**	5463.8 ±1097.6	6089.5 ±1115.3	960.9 ± 513.3	655.6 ± 391.2
**15 min**	5477.5 ±1118.4	6148.4 ±1172.5	959.7 ± 517.2	684.4 ± 378.9
**20 min**	5461.2 ±1074.0	6295.4 ±1189.2	958.9 ± 498.1	674.2 ± 368.3
**25 min**	5395.4 ±1024.8	6281.9 ±1246.9	1010.4 ± 493.7	658.7 ± 380.8
**30 min**	5445.0 ±1100.3	6315.4 ±1194.8	1031.7 ± 498.9	682.7 ± 371.4

**Table 5:** Time-dependent changes in sensor area and center of back support pressure locations according to the wheelchair design (RX-ABS Lo and RX-1).

	Sensor area of back support (cm^2^)		Center of back support pressure (top to bottom) (mm)		Center of back support pressure (right to left)	
	RX_ABS Lo	RX-1	RX_ABS Lo	RX-1	RX_ABS Lo	RX-1
**0 min**	227.6 ± 76.6	112.7 ± 52.9	194.5 ± 65.1	208.1 ± 73.5	33.4 ± 12.3	28.6 ± 32.3
**5 min**	198.3 ± 86.1	105.9 ± 56.9	178.2 ± 67.7	201.7 ± 74.6	31.4± 15.1	32.3 ± 16.1
**10 min**	178.9 ± 85.5	113.6 ± 52.3	167.7 ± 69.	189.1 ± 71.2	32.6 ± 18.4	28.0 ± 16.0
**15 min**	177.6 ± 89.6	118.9 ± 46.9	164.6 ± 73.0	196.8 ± 75.9	33.6 ± 19.8	31.0 ± 14.8
**20 min**	176.8 ± 86.2	119.7 ± 44.4	157.6 ± 75.5	191.7 ± 75.1	39.2 ± 25.4	36.4 ± 20.6
**25 min**	186.7 ± 83.2	115.8 ± 49.5	152.5 ± 74.3	194.6 ± 74.1	34.0 ± 22.9	31.5 ± 15.5
**30 min**	190.5 ± 83.3	118.7 ± 41.2	154.0 ± 73.9	176.7 ± 76.4	38.0 ± 22.0	38.4 ± 24.5

**Table 6:** Time-dependent changes in the center of buttock pressure location according to the wheelchair design (RX-ABS Lo and RX-1).

	Sensor area of buttock pressure (cm^2^)		Center of buttock pressure location (right to left, mm)		Center of buttock pressure location (forward to backward, mm)	
	RX_ABS Lo	RX-1	RX_ABS Lo	RX-1	RX_ABS Lo	RX-1
**0 min**	118.1 ± 26.0	131.3 ± 28.5	33.7 ± 76.6	29.2 ± 14.2	193.0 ± 62.2	217.0 ± 60.4
**5 min**	117.3 ± 23.9	128.1 ± 29.1	33.9 ± 14.8	30.7 ± 14.7	192.6 ± 61.8	215.3 ± 60.0
**10 min**	122.7 ± 27.2	130.6 ± 27.8	35.6 ± 14.8	31.4 ± 15.5	186.3 ± 58.5	210.9 ± 58.6
**15 min**	121.4 ± 26.1	134.2 ± 24.8	36.3 ± 15.1	30.7 ± 15.6	194.5 ± 59.1	210.8 ± 60.2
**20 min**	118.6 ± 26.5	136.5 ± 26.3	38.4 ± 18.7	30.8 ± 15.8	183.4 ± 59.4	210.5 ± 59.9
**25 min**	118.0 ± 25.3	136.3 ± 25.8	36.8 ± 18.1	30.5 ± 14.7	182.6 ± 59.0	210.9 ± 60.3
**30 min**	117.6 ± 26.8	136.4 ± 24.5	37.4 ± 17.9	30.7 ± 15.5	183.2 ± 58.5	209.1 ± 59.6

### Comparison of repositioning attempts between RX_ABS Lo and RX-1

Significantly fewer repositioning attempts were observed with RX_ABS Lo than with RX-1 (1.0 ± 1.5 vs. 6.2 ± 5.1, P = 0.014, Table 7).

**Table 7:** The time of repositioning (RX-ABS Lo and RX-1).

	RX_ABS Lo	RX-1	p value
The time of repositioning	1.0 ±1.5	6.2 ±5.1	0.014

## Discussion

This study aimed to assess the utility of the wheelchair, i.e., RX_ABS Lo, in preventing FHP in disabled elderly individuals during prolonged sitting. After a sitting duration of 30 minutes, cervical angles with RX_ABS Lo were larger than those with RX-1. Moreover, cervical angles with RX_ABS Lo were more erect, indicating RX_ABS Lo has utility in preventing FHP. Sawada et al. reported that the cervical angle of healthy elderly individuals in wheelchairs incorporating pelvic support was 56.3° and significantly more erect than with that when using the standard wheelchair (50.3°) [18]. Hatta et al. performed the same experiment for individuals with hemiplegia and reported a cervical angle of 45.3° for wheelchairs incorporating pelvic support, which is significantly more erect than that when using the standard wheelchair (40.6°) [30, 37]. These reports indicate that wheelchairs incorporating pelvic support improves FHP in the short term [18, 30, 37]. However, individuals with physical dysfunction often use wheelchairs for daily activities. In recent studies of prolonged sitting, the majority of individuals were observed while sitting in chairs with back supports [22, 27, 35]. Although a number of studies have assessed postural changes occurring in wheelchair users over durations greater than 90 minutes, the individuals involved in these studies were all healthy [30, 37]. Accordingly, we believe the present study has particular clinical significance as it is the first to assess the utility of wheelchairs incorporating pelvic supports in improving FHP in elderly disabled individuals during prolonged sitting [38,39].

Cervical angles with RX_ABS Lo did not significantly differ over the 30 minutes of sitting. However with RX-1, cervical angles were smaller after 30 minutes than 0 minutes values. Moreover, a greater number of repositioning attempts were observed with RX-1 than with RX_ABS Lo. These data indicate that the use of RX-1 exacerbated FHP because prolonged sitting increases the backward tilting of pelvis [40].

### Improvement of FHP using RX_ABS Lo

The back support angle of RX_ABS Lo is 106°, increasing to 130° in the region of the handle. Therefore, cervical angles are greater with RX_ABS Lo than with RX-1. Moreover, the thoracic and pelvic support belts were applied in shape of an arc according to the “active balance seating theory” to attain a supportive angle[16]. The beginning cervical angle with RX_ABS Lo was approximately 36°, with a more erect head and neck alignment compared to RX-1. These results indicate that RX_ABS Lo prevented FHP in the individuals.

In an ideal posture alignment, flexion along the axis of the cervical spine in relation to the head’s center of gravity is minimized[17]. When the head and neck are erect, it is possible to sit balanced using minimal strength [24, 41, 42], thereby reducing the need for repositioning.

Ergonomic studies have reported that the support of the thoracic and lumbar regions can improve posture. Three methods to attain this have been reported: the first method uses a protruding lumbar region of the back support [21], the second uses the placement of a pillow at lumbar spine L3 level and the third one uses the adjustment of lumbar support width by approximately 5 cm to prevent posterior pelvic rotation [43, 44].

Each of these supports provides a sitting posture with minimal back muscle load for chair or wheelchair users [15, 43, 44]. The prevention of posterior pelvic rotation using simple planar lumbar supports maintains the lordosis of the lumbar spine [45, 46]. Although adding simple planar lumbar support to back supports has been shown to improve anterior pelvic rotation and trunk inclination, it was not shown to improve FHP [21, 47]. To improve FHP for wheelchair uses, a pelvic support belt and an inclined back support are required [43]. With RX-1, the back support contact area was narrow with a short length of lateral back support contact. This result indicates that the RX-1 back support cannot provide adequate thoracic kyphosis and rib cage support. RX-1 provides little thoracic support, except for the inclined back support [15, 48]. The trunk angle with RX_ABS Lo was approximately 110°, and individuals had significantly greater posterior trunk rotation when compared with RX-1. Moreover, the total pressure and sensor area of the RX_ABS Lo back support were larger than those of the RX-1 back support. The lateral length of the back support contact area of RX_ABS Lo was also larger than that of RX-1. These results demonstrated that the rounded support belt that stretched from the back support of RX_ABS Lo provided adequate rib cage support.

## Limitations

One of the inclusion criteria of this study is that the individuals understood the purpose of this research. Since we used a small sample size, our results are generalizable only to a limited population. In addition, although FHP is affected by kyphosis, maintaining standing or sitting position is often challenging. Hence, we were unable to measure the degree of kyphosis and thereby directly evaluate the association between FHP and kyphosis.

Also, we could not collect data about users comfort and satisfaction. Therefore, we could not discuss about the relation between posture and comfort.

## Conclusions

We demonstrate that a wheelchair incorporating pelvic support has utility in improving FHP in elderly disabled individuals during prolonged sitting. Although the sample size of the present study was small, the findings may be useful for further studies on other populations using wheelchairs, including RCTs. The results of this study contribute to the decision-making process of consumers, clinicians, and manufacturers regarding the choice of a wheelchair seating system.
